# Growth performance and physiometabolic safety of probiotic–Curcuma herbal supplementation in local female lambs of Indonesia

**DOI:** 10.14202/vetworld.2026.964-977

**Published:** 2026-03-12

**Authors:** Yanuartono Yanuartono, Alfarisa Nururrozi, Soedarmanto Indarjulianto, Alsi Dara Paryuni, Dwi Sunu Datrianto, Imron Rosyadi, Hary Purnamaningsih

**Affiliations:** 1 Department of Internal Medicine, Faculty of Veterinary Medicine, Universitas Gadjah Mada, Yogyakarta, Indonesia; 2 Department Clinical Pathology, Faculty of Veterinary Medicine, Universitas Gadjah Mada, Yogyakarta, Indonesia

**Keywords:** *Curcuma longa*, growth performance, herbal supplementation, lamb, physiometabolic safety, probiotic supplementation, ruminant nutrition, weight gain

## Abstract

**Background and Aim:**

The escalating demand for animal protein in Indonesia underscores the imperative for efficacious nutritional strategies to augment lamb productivity while safeguarding physiological integrity. Prior investigations have predominantly examined probiotics or herbal additives in isolation, yielding fragmented insights into their combined efficacy. This study endeavored to assess the integrated impacts of probiotic–herbal supplementation on growth performance, hematological indices, and serum biochemical profiles in local Indonesian lambs maintained on a concentrate-based ration, thereby furnishing comprehensive evidence on growth dynamics and physiometabolic safety.

**Materials and Methods:**

Twelve female local lambs, aged approximately 6-7 months with an initial body weight (BW) of 20.33 ± 2.24 kg, were allocated into two cohorts: a control group receiving solely the basal diet and a treatment group administered the basal diet supplemented with a probiotic–herbal formulation. The supplement comprised *Lactobacillus* sp. (1.00 × 10^7^ CFU/mL), *Aspergillus* sp. (1.00 × 10^5^ CFU/mL), *Saccharomyces cerevisiae* (5.25 × 10^7^ CFU/mL), and *Azotobacter* sp. (8.20 × 10^6^ CFU/mL), amalgamated with *Curcuma longa* and *Curcuma xanthorrhiza* (Herbal Farm, Sido Muncul, Semarang, Indonesia). The formulation was dispensed daily at 10 mL/L in drinking water over a 4-week duration following a 1-week acclimatization phase. BWs were quantified weekly utilizing a YDTech TCS-300 digital balance (Hangzhou Sifang Electronic Scales Co., Hangzhou, China). Blood specimens were procured post-treatment via jugular venipuncture into ethylenediaminetetraacetic acid-anticoagulated tubes for hematological evaluation using a Sysmex automated analyzer (Sysmex Corporation, Hyogo, Japan), encompassing red blood cell count, hemoglobin, and packed cell volume (PCV). Serum aliquots were analyzed for lipid profiles (total cholesterol, triglycerides, high-density lipoprotein [HDL], low-density lipoprotein [LDL]), hepatic enzymes (alanine aminotransferase [ALT], aspartate aminotransferase [AST], alkaline phosphatase [ALP], gamma-glutamyl transferase), and protein fractions (total protein, albumin [ALB], globulin [GLOB], ALB:GLOB ratio) employing Biocheck kits (Biocheck, Reeuwijk, Netherlands). Clinical metrics, including body temperature, pulse rate, respiratory rate, and rumen motility, were monitored weekly. Data underwent Shapiro-Wilk normality assessment followed by Student’s t-test for intergroup comparisons (p < 0.05) via JMP software version 18 (SAS Institute Inc., North Carolina, USA). Ethical oversight was provided by the Research Ethics Committee of the Faculty of Veterinary Medicine, Universitas Gadjah Mada (approval 111/EC-FKH/Int./2025).

**Results:**

Clinical parameters in both cohorts resided within physiological norms, with the treatment group manifesting enhanced stability in body temperature, pulse rate, respiratory rate, and rumen motility relative to controls. Supplementation elicited a significant elevation in weekly weight gain (1.13 ± 0.63 kg versus 0.67 ± 0.31 kg; p = 0.00177), albeit final BWs were comparable (21.99 ± 2.59 kg versus 21.39 ± 2.99 kg; p > 0.05). Hematological indices (red blood cell: 8.86 ± 1.65 × 10¹²/L versus 9.90 ± 2.09 × 10¹²/L; hemoglobin: 9.07 ± 1.04 g/dL versus 9.94 ± 1.78 g/dL; PCV: 28.67 ± 4.88% versus 26.44 ± 5.84%) remained unaltered and normative. Lipid analyses disclosed diminished triglycerides (42.50 ± 33.61 mg/dL versus 65.67 ± 56.53 mg/dL; p = 0.049) and LDL (14.67 ± 6.41 mg/dL versus 23.71 ± 14.08 mg/dL; p = 0.037) in treated lambs, with total cholesterol and HDL unaffected. Hepatic enzymes and protein profiles exhibited no intergroup disparities, though ALP and AST surpassed conventional ranges in both, indicative of physiological adaptation rather than pathology.

**Conclusion:**

Probiotic–herbal supplementation incorporating *C. longa* and *C. xanthorrhiza* augments growth performance and ameliorates lipid metabolism in local lambs sans deleterious impacts on clinical, hematological, or hepatic profiles. These outcomes advocate its utility as a sustainable, antibiotic-alternative strategy for enhancing ruminant productivity and metabolic resilience in tropical contexts.

## INTRODUCTION

The demand for animal protein in Indonesia continues to increase. Among the livestock species that contribute to this demand, lamb plays an essential role as a source of animal protein. Through the Directorate General of Livestock and Animal Health Services, the Indonesian Ministry of Agriculture has been actively promoting the strategic development of the lamb and goat industry. In 2021, the national lamb population reached 17.9 million, representing an increase of 379,302 from the previous year. Concurrently, the goat population rose to 19.3 million, an increase of 539,356 from the previous year. Health and nutritional management exert a substantial influence on animal performance [1, 2], with feed constituting the predominant cost factor in ruminant production systems.

Probiotic supplementation strategies have been extensively investigated to enhance productivity and health status in lambs. Probiotics have been demonstrated to improve weight gain, overall health performance, metabolic activity, and immune modulation in ruminants [3–5]. Furthermore, probiotics modulate blood parameters, rumen fermentation patterns, and general productivity outcomes [6, 7]. Consequently, alternative approaches are actively explored to optimize animal feeding regimens, mitigate ruminal disorders, augment performance metrics, and improve economic returns.

In recent years, research emphasis has shifted toward combined probiotic–herbal supplementation to enhance feed efficiency and minimize chemical residues in animal-derived products [8]. These natural additives are regarded as safer and more sustainable substitutes for antibiotic growth promoters (AGP) [1]. Herbal agents such as *Curcuma longa* (turmeric), *Curcuma xanthorrhiza* (Javanese turmeric), *Zingiber officinale* (ginger), and *Allium sativum* (garlic) contain diverse bioactive constituents, including alkaloids, saponins, terpenes, and glycosides, that may positively influence animal performance and health status [2, 8].

The concurrent application of *Lactobacillus* sp., *Aspergillus* sp., *Saccharomyces cerevisiae*, and *Azotobacter* sp. with curcuma-based herbs is mechanistically underpinned by their complementary and synergistic roles within the rumen–host axis. *Lactobacillus* sp. supports microbial equilibrium and immune modulation, whereas *S. cerevisiae* and *Aspergillus* sp. augment rumen fermentation through enhanced fiber degradation, rumen pH stabilization, and elevated volatile fatty acid production [1, 2, 7, 8, 9]. *Azotobacter* sp. facilitates nitrogen utilization via biological nitrogen fixation and promotion of microbial protein synthesis. *Curcuma*-based herbs, enriched in curcumin, confer antimicrobial, antioxidant, and anti-inflammatory properties and may serve as fermentable substrates that improve probiotic viability and functional activity [10].

Despite these theoretical advantages, empirical investigations confirming the safety and potential physiological adverse effects of combined probiotic–herbal supplementation remain limited. Most extant studies in lambs have primarily emphasized productivity-oriented endpoints, such as feed efficiency and weight gain, while overlooking comprehensive assessments of physiometabolic safety [3, 11, 12]. For instance, although individual evaluations of probiotics or herbal additives have demonstrated beneficial effects on rumen fermentation and immune function [13–17], the synergistic interactions in combined regimens, particularly those incorporating underexplored herbs like *C. xanthorrhiza*, have not been systematically examined for potential risks, including alterations in hematological profiles, hepatic enzyme activities, or lipid metabolism [18–20]. This gap is particularly pronounced in region-specific contexts, such as Indonesian local sheep breeds, where environmental factors, genetic predispositions, and traditional farming practices may modulate responses to supplementation [21–23]. Moreover, the reliance on antibiotic alternatives in tropical ruminant systems necessitates rigorous safety profiling to ensure no inadvertent impacts on systemic health, microbial dysbiosis, or long-term productivity [24–26]. Consequently, there exists a critical need for integrated studies that concurrently evaluate growth performance alongside multifaceted health parameters to validate the safety and efficacy of probiotic–herbal combinations in small ruminants.

Therefore, this study aimed to elucidate the effects of a combined probiotic–herbal supplementation regimen on growth performance, clinical physiology, hematological indices, and serum biochemical profiles in local female lambs of Indonesia fed a concentrate-based diet. Specifically, we sought to quantify changes in body weight (BW) gain, rumen motility, and vital signs while assessing hematological parameters (e.g., red blood cell count, hemoglobin concentration, and packed cell volume [PCV]) and biochemical markers encompassing lipid profiles (total cholesterol, triglycerides, high-density lipoprotein (HDL), and low-density lipoprotein [LDL]), hepatic function enzymes (alanine aminotransferase [ALT], aspartate aminotransferase [AST], alkaline phosphatase [ALP], and gamma-glutamyl transferase [GGT]), and protein fractions (total protein [TP], albumin [ALB], globulin [GLOB], and ALB:GLOB ratio). By employing a randomized experimental design with control and treatment groups, this investigation intended to provide empirical evidence on the physiometabolic safety of a formulation comprising *Lactobacillus* sp., *Aspergillus* sp., *S. cerevisiae*, and *Azotobacter* sp. integrated with *C. longa* and *C. xanthorrhiza*, thereby addressing the paucity of data on combined supplementation in tropical lamb production systems. Ultimately, the findings are anticipated to inform sustainable nutritional strategies that enhance productivity without compromising animal welfare or health integrity, contributing to region-specific guidelines for Indonesian livestock management.

## MATERIALS AND METHODS

### Ethical approval

Ethical approval for this study was granted by the Research Ethics Committee of the Faculty of Veterinary Medicine, Universitas Gadjah Mada, Yogyakarta, Indonesia, under approval protocol number 111/ECFKH/Int./ 2025. All research procedures were conducted in compliance with international guidelines and standards for animal welfare.

### Study period and location

The study was conducted using a completely randomized design from June to August 2025 at Merapi Farm, Cangkring Malang Village, Sleman Regency, Yogyakarta Province, Indonesia.

### Experimental design

**Figure 1:**
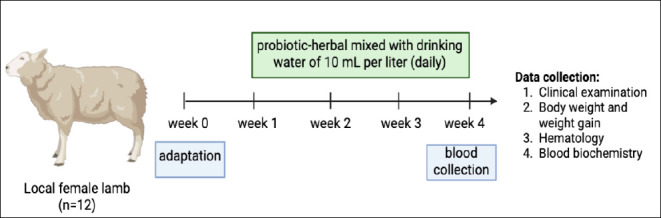
Research protocol and data collection schedule. The lambs were clinically examined and dewormed, followed by a 1-week adaptation period. A combined probiotic–herbal supplement was administered daily at a dosage of 10 mL per liter of drinking water. Data collection was performed weekly and included clinical examinations, body weight measurements, and blood sample collection.

Twelve local female lambs, approximately 6-7 months old and weighing 20.33 ± 2.24 kg, were used in this experiment. The number of lambs used in this study was determined by carefully balancing scientific objectives with ethical considerations. The sample size selection followed the principles of animal welfare and the 3Rs (Replacement, Reduction, and Refinement), prioritizing the use of the minimum number of animals necessary to achieve valid and interpretable results. The sample size was aligned with statistically comparable studies reported in the literature and was approved in accordance with institutional ethical clearance requirements. Each lamb was housed individually in a raised housing system with slatted flooring, measuring 1.5 m in length and 1.0 m in width per pen, to allow controlled feeding and supplementation. All lambs underwent a thorough clinical examination and deworming therapy using albendazole 5 mg/kg BW before the experiment to ensure the absence of endoparasitic infections. All lambs underwent a 1-week acclimatization period before the treatment phase and were subsequently divided randomly into two groups, each consisting of six animals. The environmental conditions within the housing facilities were maintained at an ambient temperature of 22°C–30°C with a relative humidity of 65%–80%. The animals were managed under a controlled feeding regimen, with feed provided twice daily and ad libitum access to well water as the sole drinking source. The pens were equipped with a manually operated ventilation system, and no vaccinations were administered during the experimental period. BWs were recorded weekly in the morning over a four-week experimental period, and blood samples were collected after the final treatment. The experimental protocol is illustrated in Figure 1.

### Feeding and drinking

The control group received a self-mixed feed (home-produced feed, uncommercially) provided by Merapi Farm. The treatment group was fed the same self-mix feed supplemented with a combination of probiotic–herbal additives (Herbal Farm, Sido Muncul, Semarang, Indonesia). The probiotic–herbal supplement was developed as a liquid preparation and administered once daily by mixing it thoroughly with drinking water at a concentration of 10 mL/L. Each lamb received 800 g of the commercial diet per day, divided into two equal portions, and clean drinking water was provided ad libitum. The nutrient composition of the feed used in this study is presented in Table 1.

**Table 1:** The proximate analysis results of the basal diet’s nutrient composition.

Basal diet composition	Percentage
Dry matter (DM)	87.92
organic matter	8.02
crude protein	16.96
crude fat	3.12
crude fiber	17.89
nitrogen-free extract	52.91

The supplement was administered once daily in the morning at 07.00 by thoroughly mixing it into the drinking water and provided until completely consumed. Thereafter, the lambs in the treatment group were given plain drinking water similar to that in the control group. The basal feed was subsequently offered at 08.00. Table 2 presents the composition of the probiotic–herbal supplement used in the treatment group.

**Table 2:** Component composition of the probiotic–herbal supplements administered.

Probiotic–herbal composition	Concentration (CFU/mL)
*Lactobacillus* sp.	1.00 × 10^7^
*Aspergillus* sp.	1.00 × 10^5^
*Saccharomyces cerevisiae*	5.25 × 10^7^
*Azotobacter* sp.	8.20 × 10^6^
Turmeric (*Curcuma longa*)	–
Javanese ginger (*Curcuma xanthorrhiza*)	–

### Clinical examination

All the lambs (n = 12) underwent weekly clinical examinations, which included body temperature, pulse, respiratory rate, and rumen motility measurements. Rectal temperature was measured using a digital clinical thermometer, and pulse rate was determined from the saphenous artery. The respiratory rate was recorded as the number of inhalations and exhalations during 60 s. The rumen motility rates were evaluated first on the left flank of each animal. All observers underwent standardized training and calibration prior to data collection to ensure inter-observer consistency.

### Analysis of BW and weight gain

The lamb’s BW was recorded weekly using a YDTech TCS-300 digital balance (Hangzhou Sifang Electronic Scales Co., Hangzhou, China), which has a capacity of 300 kg and a precision of 2 g. The total weight gain (TWG) was calculated as the difference between the final and initial BW.

### Blood samples

Blood samples (5 mL) were collected from the jugular vein of each lamb before supplementation and daily feeding. The samples were carefully transferred into commercial glass tubes containing ethylenediamine tetraacetic acid as an anticoagulant to prevent hemolysis. The complete blood count was determined using an automated hematology analyzer (Sysmex Corporation, Hyogo, Japan) to measure red blood cells (RBC), PCV, and hemoglobin (Hb) concentration. Serum was obtained by centrifugation of whole blood, and the separated fractions were stored at −20°C until biochemical analyses were conducted. All samples were aliquoted before storage, and repeated freeze–thaw cycles were avoided to maintain sample integrity. Biochemical parameters were analyzed using commercially available reagents and kits (BioCheck, Reeuwijk, Netherlands) and included the following: 1. lipid profile: total cholesterol, triglycerides, HDL, and LDL; 2. liver function parameters: AST, ALT, ALP, and GGT; and 3. protein profile: TP, ALB, GLOB, and ALB-to-GLOB (A/G) ratio.

### Statistical analysis

Data obtained from clinical examinations were analyzed descriptively, whereas BW, TWG, hematological, and biochemical parameters were analyzed quantitatively. Data collection and outcome assessment were conducted under blinded conditions to minimize potential sources of bias. Quantitative data were statistically compared using parametric tests following the assessment of normality and homogeneity using the Shapiro-Wilk test. When the data met the assumption of normality and homogeneity, comparisons between groups were conducted using Student’s t-test. A p-value of 0.05 was considered statistically significant. All statistical analyses were performed using the JMP software version 18 (SAS Institute Inc., North Carolina, USA).

## RESULTS

### Clinical examination

The clinical examination (Table 3) indicated that both groups exhibited physiological parameters within the normal range, despite showing minor fluctuations during the observation period. The control group exhibited a gradual decline in temperature throughout the observation period. In contrast, the treatment group maintained a relatively stable body temperature across all weeks. The control group exhibited a gradual decline in pulse rate from the first to the final week, indicating a mild reduction in circulatory activity, whereas the treatment group maintained a steady pulse rate with only slight variations. In the control group, a gradual downward trend in respiration rate was observed from weeks 1 to 4, whereas the treatment group maintained relatively stable values with only minor fluctuations throughout the observation period. As the experiment progressed, the control group showed a mild reduction in the frequency of rumen movements, which became particularly evident by week 4. In contrast, the treatment group maintained consistent rumen contractions from weeks 1 to 4, showing only slight variations across the observation period. During the initial weeks (1–2), both groups exhibited similar rumen movement values, suggesting comparable digestive function at baseline.

**Table 3:** Clinical presentations of lamb physical examinations at the individual level (n = 12).

Clinical parameter [9–11]	Group	Average	Week 1	Week 2	Week 3	Week 4
Temperature (°C)	Control	39.7	39.4	39.2	37.8	
	Treatment	39.8	39.4	39.6	39.4	
Pulse rate (per minute)	Control	126	118	107	106	
	Treatment	121	116	95	104	
Respiration rate (per minute)	Control	62	74	57	50	
	Treatment	58	72	60	68	
Rumen motility rate (in mL)	Control	2	3	2	2	
	Treatment	2	4	2	2	

### BW and TWG

The final average BW (Table 4) measurements showed no significant differences between the groups. However, the average TWG of the treatment group was significantly higher than that of the control group (p < 0.05).

**Table 4:** Final average body weight (BW) measurements of experimental lamb (n = 12).

Parameter	Average BW (kg)		p-value

	Control	Treatment	
BW	21.39 ± 2.99	21.99 ± 2.59	>0.05
Total weight gain	0.67 ± 0.31	1.13 ± 0.63	0.00177

### Blood hematology

The mean values of RBC, PCV, and hemoglobin in both groups were within the reference physiological ranges defined for the corresponding species and age category (Table 5). No statistically significant differences in RBC count, Hb levels, and PCV were observed between the groups.

**Table 5:** Effects of probiotics on lamb blood hematology (n = 12).

Parameter	Normal range [12]	Control	Treatment	p-value
RBC (10¹²/L)	9.00 - 15.80	9.90 ± 2.09	8.86 ± 1.65	>0.05
PCV (%)	27.00 - 45.00	26.44 ± 5.84	28.67 ± 4.88	>0.05
Hb (g/dL)	9.0 - 15.0	9.94 ± 1.78	9.07 ± 1.04	>0.05

RBC = Red blood cells, PCV = Pack cell volume.

### Blood biochemistry

The results of the lipid profile analysis (Table 6) revealed an increase in triglyceride (TG) levels that exceeded the expected physiological range in both the control and treatment groups. Additionally, statistically significant differences were observed in TG and LDL concentrations between the treatment and control groups. The treatment group supplemented with probiotics exhibited markedly lower TG and LDL levels than the control group. The HDL concentration did not significantly differ between the control and treatment groups, and both values remained within the normal physiological range for the lamb. Although no significant difference was detected in total cholesterol levels between the control and treatment groups, the treatment group exhibited a lower mean total cholesterol concentration than the control group. Nevertheless, both values were within the normal reference range for lamb.

**Table 6:** Blood biochemistry examination results included lipid profiles, liver function parameters, and protein profiles (n = 12).

Parameters	Normal range [12]	Control	Treatment	p-value
**Lipid profiles**				
Total cholesterol (mg/dL)	52-76	71.88 ± 25.49	62.08 ± 18.17	>0.05
Triglycerides (mg/dL)	10-30	65.67 ± 56.53	42.50 ± 33.61	0.049*
HDL (mg/dL)	30-50	34.58 ± 11.38	36.25 ± 8.53	>0.05
LDL (mg/dL)	10-25	23.71 ± 14.08	14.67 ± 6.41	0.037*
**Liver function**				
ALT (U/L)	45-80	101.92 ± 60.12	106.58 ± 48.62	>0.05
AST (U/L)	60–280	99.50 ± 33.93	93.08 ± 32.49	>0.05
ALP (U/L)	59–300	297.29 ± 76.84	271.04 ± 106.48	>0.05
GGT (U/L)	20–52	34.71 ± 11.94	38.50 ± 15.57	>0.05
**Protein profiles**				
TP (g/dL)	6.0–7.9	6.91 ± 1.12	6.67 ± 1.11	>0.05
ALB (g/dL)	2.4–3.6	3.66 ± 0.67	3.60 ± 0.60	>0.05
GLOB (g/dL)	3.0–5.4	3.25 ± 0.72	3.08 ± 0.90	>0.05
Ratio A:G	0.6 - 1.5	1.16 ± 0.29	1.45 ± 1.40	>0.05

### Liver function

In this study, liver function, including ALT, AST, ALP, and GGT, showed no statistically significant differences between the control and treatment groups (Table 6). However, the mean ALP and ALT values in both groups were elevated above the normal physiological range. The ALP levels in the control and treatment groups were higher than the reference range of 30-130 U/L. Similarly, the ALT concentration increased in both groups, exceeding the normal range of 45-80 U/L.

### Protein profile

Blood biochemical analysis evaluating TP, ALB, GLOB, and the A:G ratio revealed no statistically significant differences between the control and treatment groups. Furthermore, all measured TP, ALB, GLOB, and A:G ratio values remained within the normal physiological range (Table 6).

## DISCUSSION

### Overall findings

Overall, the present findings demonstrate that combined probiotic and curcuma-based herbal supplementation improved growth performance in local lambs without causing any adverse physiological, hematological, or biochemical effects. All clinical parameters stayed within normal physiological ranges throughout the experimental period, confirming that the supplementation was well tolerated. The lack of significant changes in hematological indices, liver enzyme activities, and protein profiles provides further evidence of the physiometabolic safety of this combined regimen. In addition, the observed improvements in lipid metabolism, particularly the reductions in triglyceride and LDL concentrations, indicate beneficial systemic effects while preserving overall physiological stability.

### Physiological parameters

Physiological parameters such as body temperature, pulse rate, respiration rate, and rumen motility serve as key indicators of animal health and adaptation to environmental or metabolic challenges. In this study, all measured values remained within normal physiological ranges, showing that supplementation had no detrimental effects. Body temperature, influenced by environmental conditions, physiological status, and genetic factors [10], was comparable between groups during the first two weeks. By week 3, the treatment group maintained a slightly higher and more stable temperature, while the control group showed a decline by week 4, suggesting improved thermoregulatory stability in the supplemented lambs. This pattern is consistent with previous reports that probiotics enhance stress resistance and physiological resilience, especially under elevated ambient temperatures [11, 12]. Pulse rates were similar between groups during the initial two weeks, reflecting comparable baseline conditions. From week 3 onward, the treatment group showed a slightly higher but stable pulse rate, whereas the control group displayed greater variability. Importantly, all values remained within normal physiological limits, indicating no cardiovascular dysfunction. Given the close relationship between pulse rate, thermal load, and metabolic demand [10, 13], this stability aligns with evidence that probiotic supplementation, particularly involving S. cerevisiae, may provide cardioprotective effects by reducing stress-related increases in heart rate [14]. Respiratory rates also remained within physiological ranges throughout the study. Both groups exhibited similar rates during the first two weeks, with only minor divergence later as the treatment group maintained greater stability. This finding is in line with earlier reports showing that probiotics can modulate immune responses and reduce inflammation, thereby supporting respiratory homeostasis under stressful conditions [15–17]. Rumen motility, a critical indicator of digestive efficiency, stayed within normal physiological limits in both groups. Probiotic supplementation is known to improve rumen fermentation, alter microbial composition, and enhance motility through favorable changes in the rumen environment [18–21]. Environmental factors, such as increased forage moisture during the rainy season, can affect rumen pH and contraction patterns [22]. A slight decline in motility appeared in the control group by week 3, whereas the treatment group maintained consistent rhythmic contractions, with clearer differences emerging by week 4. The sustained rumen motility in the supplemented lambs suggests improved rumen stability, more efficient fermentation, and better nutrient utilization, ultimately contributing to enhanced feed efficiency and productivity in ruminants [20, 21, 23].

### Rumen–host axis and systemic implications

Probiotics are increasingly recognized as functional feed additives that play a central role in maintaining gastrointestinal ecological balance, with effects extending beyond local digestive processes. They help stabilize rumen microbial communities and rumen motility by competing for nutrients, producing antimicrobial compounds, and colonizing specific niches along the gastrointestinal mucosa, thereby supporting rumen homeostasis [13–15]. This stabilized rumen environment is now understood as a key determinant of systemic physiological regulation, as evidenced by consistent physiological parameters and immune responsiveness. Accordingly, indicators such as sustained motility and microbial equilibrium can serve as indirect markers of the functional link between rumen and systemic health. Within this framework, probiotic supplementation contributes not only to improved growth efficiency but also to enhanced systemic resilience and disease resistance, highlighting its broader importance in ruminant health management [16, 17].

### Regional and contextual relevance

Research on this specific combination in small ruminants, particularly local female lambs, remains limited, as most previous studies have evaluated probiotics and herbs separately. Probiotic–herbal supplementation has been more widely applied in Indonesian poultry, cattle, and goats [18–20]. The combination of probiotics and herbs has been proposed as an alternative feed strategy to improve dry matter intake, feed efficiency, and nutrient utilization [2]. Furthermore, this approach may partially replace antibiotics by suppressing pathogenic bacteria, stimulating immune responses, and reducing methane production, thereby minimizing energy loss. Collectively, these mechanisms support improved livestock growth performance and overall health. Since 2017, Indonesia has banned the use of AGP in livestock feed under Minister of Agriculture Regulation No. 14/2017 to combat antimicrobial resistance and reduce antibiotic residues in animal-derived products. Probiotics and herbal additives have since been widely adopted as alternative feed supplements in livestock production. Demonstrating that probiotic–herbal supplementation enhances immune function and overall health can support national efforts to reduce the emergence of antibiotic resistance linked to livestock systems. Native Indonesian lambs were chosen for this study due to their proven adaptability to tropical environments and year-round reproductive capacity. Although local lambs typically exhibit small body size, slow growth rates, coarse wool, and relatively low meat yield, they represent a valuable genetic resource for sustainable smallholder livestock systems in Indonesia. The exclusive use of local female lambs is especially relevant, as their continuous reproductive ability throughout the year offers considerable potential for improving population growth and production efficiency. Accordingly, this study provides original evidence specific to local breeds, addressing a critical gap in region-relevant livestock research.

### BW

In this study, probiotic–herbal supplementation resulted in greater BW gain compared with the control group. Several mechanisms may explain this improvement. Du *et al*. [21] reported that probiotic–herbal administration stabilized gastrointestinal microbiota in growth-retarded calves, thereby stimulating growth and enhancing host defense mechanisms. Wang *et al*. [22] further showed that probiotics containing *Aspergillus* sp., similar to those used here, improved rumen function in Hu lambs by increasing villus length and jejunal crypt depth, facilitating greater nutrient absorption. Additionally, Arowolo and He [2] noted that S. cerevisiae-based probiotics enhance fiber digestion, increasing the availability of structural carbohydrates such as cellulose and hemicellulose as energy sources for ruminants. The herbal supplement in this study contained curcumin as its primary active compound, which has been reported to improve the digestibility of neutral detergent fiber and nitrogen, both essential for enhancing nutrient absorption and growth performance [4, 23]. Curcumin supplementation has also been linked to improved energy and nitrogen metabolism, contributing to better growth efficiency. Moreover, curcumin increases serum activity of antioxidant enzymes such as superoxide dismutase and glutathione peroxidase thereby reducing oxidative stress [24, 25]. This antioxidant effect has been consistently observed across studies, reflecting a strengthened antioxidant defense system in lambs [26].

### Blood hematology

Probiotic–herbal supplementation did not induce any notable alterations in hematological parameters. The absence of changes can be considered beneficial, as it indicates that the supplementation did not cause hematological disorders such as anemia, hemolysis, or erythrocytosis. A similar study by Zeitoun *et al*. [27] on Noemi lambs also reported no significant differences in hematological profiles following herbal supplementation. These findings suggest that the management and care practices used had a stabilizing effect, while also highlighting the limited research on the influence of probiotic-derived fiber on hematological traits in lambs [5].

### Blood chemistry

In the present study, probiotic–herbal supplementation reduced triglyceride and LDL levels, while liver function and protein profiles remained within normal ranges. Saravani *et al*. [28] similarly reported a significant decrease in total cholesterol in Zeel lambs supplemented with a probiotic–herbal mixture containing *Cuminum cyminum*, lemongrass, and Coriandrum sativum. Other studies in lambs have also demonstrated cholesterol-lowering effects of probiotic–herbal combinations, although the extent of reduction varies [29]. According to Kianbakht and Jahaniani [30], bioactive compounds in these supplements reduce cholesterol and blood lipids by producing enzymes that degrade bile acids and lower intestinal pH. The decline in serum cholesterol observed here likely results from the synergistic effects of probiotics on lipid metabolism and lipoprotein transport. As cholesterol serves as a precursor for bile acid synthesis, increased utilization for this process may explain the reduction in circulating cholesterol [31]. No significant difference in HDL levels was found between the control and treatment groups, with both values remaining within normal ranges. Similarly, Ramdani *et al*. [32] reported above-average HDL values in both groups of lambs, consistent with Kafilzadeh *et al*. [7], who found no effect of L. acidophilus, *L. casei*, *Bifidobacterium thermophilum*, and *Enterococcus faecium* supplementation on HDL in Sanjabi lambs. Differences in HDL responses across studies may arise from variations in probiotic formulation, herb inclusion, feed composition, breed, or physiological status [33]. Significant differences in triglyceride and LDL levels were observed between groups (Table 3). The control group had higher triglycerides than the treatment group, exceeding the normal range for lambs (10–30 mg/dL). These results align with those of Khattab *et al*. [34], who reported lower triglycerides in *B. subtilis*-treated Barki lambs compared with controls. The reduction in triglyceride levels observed here likely reflects decreased hepatic fatty acid synthesis [35] and enhanced lipase activity promoting fat hydrolysis [36]. As shown in Table 6, LDL levels were significantly lower in the treatment group than in the control group, although both remained within normal physiological limits. This finding is consistent with Sheikh *et al*. [37], who observed similar effects in Corriedale lambs. The LDL reduction likely results from decreased total cholesterol through probiotic-mediated bile acid deconjugation and altered cholesterol metabolism [38, 39].

### Liver function

Liver function is a critical indicator of overall animal health. Enzyme activity, including ALT, AST, ALP, and GGT, provides valuable insight into hepatic status and metabolic performance. No significant differences were observed in ALT, AST, ALP, and GGT levels between the control and treatment groups (Table 6). These findings suggest that supplementation did not adversely affect hepatic integrity or enzyme homeostasis in lambs. Similar observations have been reported by Kafilzadeh *et al*. [7], who found no significant differences in ALT, AST, or ALP activities in probiotic-supplemented Sanjabi lambs, and by Mahmoud *et al*. [23], who also demonstrated stable hepatic enzyme profiles in probiotic- and prebiotic-treated lambs. Although the overall enzyme values were statistically comparable between groups, both ALT and ALP levels were higher than standard reference ranges. Mean ALP values were 297.29 U/L in the control group and 271.04 U/L in the treatment group, exceeding the typical physiological range of 30–130 U/L. Rather than indicating hepatocellular injury, elevated ALP activity may reflect high metabolic demands associated with growth, bone development, or transient stress responses. Comparable elevations have been reported in Afshari sheep by Karbasi *et al*. [39], who observed mean ALP values of 272.6 ± 120.4 U/L, with the highest concentrations in younger animals. Similarly, ALT values of 101.92 U/L and 106.58 U/L in the control and treatment groups, respectively, also exceeded conventional limits (45–80 U/L), consistent with the wide variability documented across lamb breeds, nutritional conditions, and management systems [40–42]. The mild elevation in ALT and AST observed in both groups could be attributed to handling, weighing, and blood sampling-related stress. Previous research has shown that such stressors can transiently elevate serum enzyme activity due to mild hepatocellular or muscular strain [43].

However, the values recorded here remained well below the hepatotoxic threshold suggested by Akanmu *et al*. [44], who considered ALP levels exceeding 464 U/L indicative of liver dysfunction. Therefore, the current findings likely reflect normal physiological adaptation rather than pathological changes. Contrasting evidence from other studies indicates that probiotic supplementation can lower hepatic enzyme activity. El-Sayed and Mousa [45] observed a significant reduction in ALT and AST concentrations in Barki lambs receiving probiotics, attributed to reduced gluconeogenic activity and improved metabolic efficiency. As ALT and AST are key transaminases involved in amino acid catabolism and gluconeogenesis, reduced activity may indicate improved hepatic function and lower oxidative stress. Similarly, Hussein [46] and Darwish *et al*. [47] reported comparable reductions or stable enzyme levels following probiotic administration in lambs, reinforcing the hepatoprotective potential of probiotics under certain conditions. Although no significant differences were observed between groups, both ALT and ALP activities exceeded standard reference ranges, warranting careful interpretation. Mild elevations of these enzymes in growing lambs are frequently associated with high anabolic activity, particularly bone growth and tissue remodeling, rather than overt hepatocellular damage [48]. ALP, in particular, is strongly influenced by osteoblastic activity and age-related growth dynamics, which may explain the uniformly elevated ALP values in both groups. Similarly, slight increases in ALT may reflect transient metabolic adaptation or handling-related stress associated with repeated weighing and blood sampling, rather than compromised liver function. Enzyme activities remained well below hepatotoxic thresholds and were not accompanied by abnormalities in other liver-associated parameters, protein profiles, or clinical signs, supporting the interpretation of a physiological rather than pathological response [43, 49].

Collectively, these findings indicate that the observed elevations likely represent normal age- and management-related variation and do not suggest adverse hepatic effects of probiotic–herbal supplementation. The effects of probiotics and herbal combinations on GGT activity in lambs remain scarce. GGT is a sensitive marker of biliary integrity and oxidative stress, and its stability is an important indicator of hepatic health. In this study, GGT levels remained within the normal range and did not significantly differ between treatment groups, suggesting that the probiotic–herbal supplement maintained normal biliary function. This observation is consistent with Mallaki *et al*. [50], who found no significant changes in GGT levels in Ghezel sheep supplemented with probiotics and bovine lactoferrin. Taken together, these findings suggest that probiotic–herbal supplemen-tation exerts a neutral to mildly beneficial effect on hepatic function. The absence of significant enzyme elevation, coupled with values remaining within physiological limits, indicates that the supplement neither induces hepatocellular stress nor compromises liver function [48, 49]. Overall, the present results reinforce the safety of probiotic–herbal supplementation and support its role in maintaining metabolic and hepatic stability in lambs under experimental conditions.

### Protein profile

The evaluation of serum protein parameters provides essential insights into the nutritional and metabolic status of animals and the potential effects of dietary supplementation on hepatic and immune function. In the present study, administration of probiotics combined with herbs did not result in significant differences in TP, ALB, GLOB, or the A:G ratio between the control and treatment groups. These results suggest that probiotic–herbal supplementation maintains normal protein metabolism and does not impair hepatic synthetic capacity or protein turnover. The lack of significant differences in protein parameters observed in this study may be attributed to hepatic function stability and the absence of inflammatory responses during the supplementation period. Probiotic–herbal effects are primarily exerted through modulation of gut microbiota, enhancement of nutrient absorption, and regulation of immune balance rather than through direct alteration of hepatic protein synthesis [43, 48, 50]. Serum protein levels typically remain stable in healthy animals with optimal nutritional intake, as homeostatic mechanisms maintain equilibrium between protein synthesis and degradation. Furthermore, the absence of change in the A:G ratio suggests that the treatment did not trigger immune activation or systemic inflammation, supporting the notion that probiotic–herbal supplementation is physiologically safe and does not induce hepatic stress or immune imbalance. Collectively, these findings support the growing body of evidence that probiotic–herbal supplementation contributes to maintaining metabolic stability without disrupting essential biochemical parameters. Future research involving longer treatment durations or animals under metabolic stress may help clarify whether probiotic–herbal interactions influence protein synthesis dynamics or immunoglobulin production under different physiological conditions.

### Synergistic mechanisms

Recent studies have increasingly examined the synergistic integration of probiotics with fermented herbal supplements. Within this framework, herbal components are proposed to function not only as bioactive agents but also as fermentable substrates that support probiotic activity, thereby enhancing the bioavailability and efficacy of herbal compounds. Concurrently, probiotics contribute to the stabilization and functional optimization of the rumen ecosystem, resulting in a more resilient and efficient ruminal environment [2, 33, 51]. From a conceptual perspective, this synergistic interaction supports the notion of a coordinated rumen–microbiota–host axis, through which combined supplementation exerts effects that extend beyond additive outcomes. Consequently, probiotic–herbal combinations are frequently associated with superior improvements in growth performance, nutrient digestibility, and rumen microbial modulation compared with either supplement alone. Studies in lambs have demonstrated that herbal components may function as fermentable substrates for probiotics, thereby enhancing the bioavailability of active herbal compounds [14]. In turn, probiotics support the stabilization and functional efficiency of the rumen environment [13]. Within this synergistic framework, the combined use of probiotics and herbal supplements has been shown to yield superior growth performance, digestive efficiency, and rumen microbiota modulation improvements compared with the administration of either supplement alone.

### Economic and practical implications

From an economic standpoint, the observed improvements in growth performance and physiological stability indicate that probiotic–herbal supplementation can enhance production efficiency without increasing health-related costs. Improved rumen function and metabolic profiles may contribute to better feed utilization and reduced reliance on therapeutic interventions. Although a formal cost–benefit analysis was not performed, these biological advantages highlight the practical relevance of this supplementation strategy and support the need for future studies incorporating comprehensive economic evaluations.

### Study novelty and contribution

This study is positioned primarily as a validation of the physiological and biochemical safety of combined probiotic–herbal supplementation rather than solely as a growth enhancement trial. The findings provide integrated evidence of physiometabolic safety in Indonesian local lambs by demonstrating stable clinical parameters, hematological indices, liver enzyme activities, and protein profiles alongside improved growth responses. The inclusion of C. xanthorrhiza, a comparatively underexplored phytobiotic in ruminants, further enhances the originality of this study by highlighting its complementary role within a probiotic–herbal regimen. Consistent rumen motility and stable physiological parameters were interpreted as indirect indicators of rumen–systemic health linkage, extending the contribution beyond conventional performance metrics and establishing a conceptual framework for broader host–microbiota interactions. In addition, the manuscript explicitly states its novelty by emphasizing the simultaneous assessment of growth outcomes and comprehensive safety endpoints, an approach rarely addressed in lamb studies. The farm-based experimental setting enhances practical relevance for smallholder systems, while the exclusive use of female lambs provides underreported, sex-specific baseline data. Collectively, the results are framed as foundational data supporting future microbiome- and omics-based investigations, with relevance to sustainable livestock production and a one-health perspective, thereby strengthening both the scientific and applied originality of the study.

### Limitations

Despite the favorable outcomes observed, this study has several limitations that should be acknowledged. First, the relatively small sample size may limit the statistical power of the analysis and the generalizability of the findings to broader populations of local sheep under diverse management systems. Second, the short experimental period may not have been sufficient to fully capture the long-term effects of probiotic–herbal supplementation on growth dynamics, rumen adaptation, and metabolic regulation. Third, dry matter intake was not directly measured in this study. This limitation should be acknowledged because the absence of dry matter intake data precludes the distinction between enhanced feed efficiency and increased feed consumption as the primary driver of weight gain. Future studies incorporating larger sample sizes, longer experimental durations, and direct assessment of dry matter intake are warranted to better elucidate the mechanisms underlying the observed performance responses.

## CONCLUSION

The present study demonstrates that daily supplementation with a combined probiotic–herbal formulation (containing *Lactobacillus* sp., *Aspergillus* sp., *S. cerevisiae*, *Azotobacter* sp., *C. longa*, and *C. xanthorrhiza*) administered at 10 mL/L in drinking water significantly improved growth performance in local female lambs without compromising physiological or metabolic health. Over the 4-week experimental period, the treatment group exhibited a markedly higher average TWG (1.13 ± 0.63 kg) compared with the control group (0.67 ± 0.31 kg; p = 0.00177), while final BWs remained statistically comparable. Clinical parameters (rectal temperature, pulse rate, respiratory rate, and rumen motility) stayed within normal physiological ranges in both groups, with the supplemented lambs showing greater stability, particularly in body temperature and rumen contractions during weeks 3 and 4. Hematological indices (RBC, Hb, and PCV) were unaffected and remained within reference ranges. Biochemically, the treatment group displayed significantly lower triglyceride (42.50 ± 33.61 mg/dL vs 65.67 ± 56.53 mg/dL; p = 0.049) and LDL concentrations (14.67 ± 6.41 mg/dL vs 23.71 ± 14.08 mg/dL; p = 0.037), indicating improved lipid metabolism, whereas total cholesterol, HDL, liver enzymes (ALT, AST, ALP, and GGT), and protein fractions (TP, ALB, GLOB, and A:G ratio) showed no significant intergroup differences. Although ALT and ALP values exceeded conventional reference ranges in both groups, the elevations were comparable and most likely attributable to physiological adaptation associated with rapid growth, bone remodeling, and handling stress rather than hepatic pathology.

These results carry important practical implications for smallholder sheep farming systems in tropical regions, particularly in Indonesia, where native breeds represent a valuable genetic resource but typically exhibit modest growth rates. The observed enhancement in weekly weight gain, combined with improved lipid profiles and sustained rumen motility, suggests that this locally accessible probiotic–herbal supplement can serve as a safe, cost-effective, and sustainable alternative to AGP, especially relevant following the nationwide ban on AGPs in 2017. The formulation’s apparent support for thermoregulatory stability and digestive consistency under variable ambient conditions further underscores its suitability for year-round production in smallholder settings.

The principal strengths of the study include the integrated evaluation of growth performance alongside a comprehensive battery of clinical, hematological, and biochemical safety endpoints, an approach that remains uncommon in lamb supplementation trials. The exclusive use of local female lambs provides much-needed breed- and sex-specific data, while the farm-based setting enhances the ecological and practical validity of the findings for real-world Indonesian production systems. Inclusion of *C. xanthorrhiza*, a relatively understudied phytobiotic in ruminants, adds originality to the work.

Limitations must also be acknowledged. The relatively small sample size (n = 6 per group) constrains statistical power and generalizability to larger or more diverse populations. The 4-week duration, although sufficient to detect meaningful differences in weight gain and selected metabolic markers, is too short to assess long-term effects on growth trajectory, rumen microbial adaptation, or reproductive performance. Furthermore, direct measurement of dry matter intake was not performed, preventing differentiation between improved feed efficiency and increased voluntary intake as the primary driver of the observed weight gain.

Future research should therefore prioritize larger-scale, multi-center trials with extended supplementation periods (≥12 weeks) to better characterize sustained performance benefits and potential carry-over effects into the finishing or reproductive phases. Incorporation of feed intake monitoring, rumen fluid sampling for volatile fatty acid profiles and microbial community analysis (e.g., 16S rRNA sequencing), and metabolomic or transcriptomic profiling would help elucidate the precise mechanisms underlying the observed improvements in growth and lipid metabolism. Comparative studies evaluating different inclusion levels, delivery methods (water vs feed), or combinations with other locally available herbs would further optimize formulation for cost-effectiveness and field applicability.

In conclusion, the present findings provide robust evidence that combined probiotic–herbal supplemen-tation containing *C. longa* and *C. xanthorrhiza* offers a safe, efficacious, and region-appropriate nutritional strategy to enhance productivity in Indonesian local lambs. By simultaneously improving growth performance and supporting favorable lipid metabolism without detectable adverse effects on clinical, hematological, or hepatic parameters, this approach aligns with national goals of reducing antimicrobial use while promoting sustainable small ruminant production. These results lay a solid foundation for broader adoption in tropical smallholder systems and warrant further mechanistic and longitudinal investigations to fully unlock the potential of such natural feed additives in modern ruminant nutrition.

## DATA AVAILABILITY

All the generated data are included in the manuscript.

## AUTHORS’ CONTRIBUTIONS

YAN and SI: Conceived and supervised the study. YAN, SI, AN, ADP, and DSD: Conducted the treatments and collected samples. IR and HP: Performed the hematological and biochemical analyses. YAN, SI, AN, and ADP: Drafted and revised the manuscript. All authors have reviewed and approved the final version of the manuscript.
